# Vineyard practices reduce the incidence of *Aspergillus* spp. and alter the composition of carposphere microbiome in grapes (*Vitis vinifera* L.)

**DOI:** 10.3389/fmicb.2023.1257644

**Published:** 2023-11-24

**Authors:** S. I. Testempasis, C. V. Papazlatani, S. Theocharis, P. A. Karas, S. Koundouras, D. G. Karpouzas, G.S. Karaoglanidis

**Affiliations:** ^1^Laboratory of Plant Pathology, Aristotle University of Thessaloniki School of Agriculture, Forestry and Natural Environment, Thessaloniki, Greece; ^2^Laboratory of Plant and Environmental Biotechnology, University of Thessaly, Department of Biochemistry and Biotechnology, Larissa, Greece; ^3^Laboratory of Viticulture, Aristotle University of Thessaloniki, School of Agriculture, Forestry and Natural Environment, Thessaloniki, Greece

**Keywords:** *Aspergillus* bunch rot disease, grape carposphere microbiome, agronomic practices, grape bunch density, leaf removal, irrigation

## Abstract

Going through the new transitioning era of the “European Green Deal,” the search for alternative, non-chemical, disease control methods is essential. *Aspergillus* bunch rot is considered one of the most important diseases of grapevines resulting in severe yield losses and, major qualitative deterioration of grape products due to the production of mycotoxins. We investigated, in a two-year field study, the impact of agronomic practices like defoliation to enhance grape microclimate (DF), pruning method to reduce grape bunch density (LBD), and irrigation cut-off (NIR), at three developmental stages of grapevine (Pea size berry, Veraison, and Harvest), on (i) grape composition (titratable acidity, pH, and total soluble solids), (ii) on the frequency of occurrence of *Aspergillus* on grape berries, and (iii) on the overall composition of grape carposphere microbiome. The density of *Aspergillus* on grape berries was significantly reduced by the applied management practices (DF, LBD, and NIR). Amplicon sequencing analysis showed that both the phenological stage and the agronomic practices employed (particularly NIR and DF) imposed significant changes in the α-diversity and β-diversity of the grape carposphere bacterial and fungal communities. The NIR, LBD, and DF treatments which supported lower *Aspergillus* populations, network analysis revealed negative co-occurrence patterns between *Aspergillus* and several bacterial genera (*Streptococcus, Rhodococcus*, and *Melitangium*) reported to have antifungal properties suggesting potential natural attenuation mechanisms for the control of *Aspergillus*. Overall, our study (i) showed that the application of halting of irrigation and thinning of leaves and grape bunches, reduce the occurrence of *Aspergillus* and hence the incidence of *Aspergillus* Bunch rot disease and (ii) identified preliminary evidence for interactions of *Aspergillus* with members of the epiphytic grape bacterial communities that might be involved in the suppression of *Aspergilli*, an observation which will be further pursued in following studies in the quest for the discovery of novel biological control agents.

## Introduction

Grape products account for a significant portion of global agricultural production, with the total area covered by vineyards exceeding seven (7) million hectares in 2018 (FAO-OIV, 2019). Grapevine and, in particular, grape berries support a diverse epiphytic microbial community (collectively called carposphere microbiome) whose composition shows strong regional patterns, and it is affected by cultivar., vintage, and agronomic practices (Bokulich et al., 2013; Papadopoulou et al., 2022). The grape carposphere microbiome encompasses microorganisms with a key role in downstream vinification processes (Bokulich et al., 2016; Liu et al., 2020), but also carries beneficial and pathogenic microorganisms (Pantelides et al., 2015) whose interaction with each other and their host determine grapevine health and productivity (Bettenfeld et al., 2022). The grape berry pathobiome, in particular, is composed of several opportunistic fungal pathogens belonging to *Aspergillus* spp., *Alternaria* spp., *Botrytis* spp., *Cladosporium* spp., *Fusarium* spp., *Rhizopus* spp., and *Penicillium* spp. (Abrunhosa et al., 2001; Somma et al., 2012; Kizis et al., 2014; Ferranti et al., 2018), that can infect the grape berries under favorable conditions (Somma et al., 2012).

*Aspergillus*, a dominant member of the grapevine pathobiome and microbiome, is present in all the developmental stages of grapes (Battilani et al., 2003; Zhu et al., 2021). *Aspergillus* bunch rot is a late-season disease caused by *Aspergillus* species belonging to the section *Nigri* (*A. carbonarius* and *A. niger* aggregate), and it may lead to severe quantitative and qualitative losses due to the production of potentially carcinogenic mycotoxins, such as Ochratoxin A (OTA) and Fumonisins (FB_2_ and FB_4_) (IARC, 1991, 2002) that deteriorate the quality of wine (Rousseaux et al., 2014; Zhu et al., 2021). Their capacity to cause grape berry rots is affected by a variability of biotic and abiotic factors, like variety, region, climate, agricultural practices, vineyard microclimate, and pests (Battilani et al., 2003; Chiotta et al., 2009; Passamani et al., 2012; Setati et al., 2012). *Aspergillus* bunch rot disease management relies on field prevention practices that aim to minimize any mechanical damage or wound formation on grape berries (Covarelli et al., 2012). The European Commission, as dictated by recent actions like “The European Green Deal” action and the EU Directive 2009/128/EC on the sustainable use of pesticides, promotes the use of Integrated Pest Management (IPM) and alternative methods to control plant pests and diseases (Sante, 2017). In IPM, several agronomic practices like irrigation and canopy management have been suggested as means to minimize disease development in grapevine (Tello et al., 2015; Mosetti et al., 2016; Thomidis et al., 2016; Figueiredo et al., 2020; Wurz et al., 2020). The integration of such agricultural practices is expected to influence the microclimate of berry clusters with reciprocal effects on the composition of the associated microbiome and pathobiome (Pastore et al., 2013). Most of the previous studies have investigated the impact of these agronomic practices on productivity, grape, and wine quality, and on the occurrence of various berry and foliar grapevine diseases such as Gray Mold (*Botrytis cinerea*) and Powdery Mildew (*Erysiphe necator*) (Austin and Wilcox, 2011; Pastore et al., 2013; Mosetti et al., 2016; Thomidis et al., 2016; Pertot et al., 2017b; Hed and Centinari, 2018; Wurz et al., 2020). However, little is known regarding the effect of such management practices on *Aspergillus* bunch rot disease and on the associated grape carposphere microbiome.

In this frame we aimed to evaluate the impact of a range of agronomic practices often utilized in vineyards like defoliation, berry cluster density thinning and halting of irrigation on (i) the *Aspergillus* population on grapes (culture-based approach) and on the grape carposphere (culture-independent approach) and (ii) the overall grape carposphere fungal and bacterial community at three phenological stages (Pea size berry, Veraison, and Harvest). Parallel measurements of grape berry quality parameters provided insights into potential mechanisms driving changes in the abundance and diversity of the carposphere grapevine microbiota.

## Materials and methods

### Vineyard site and experimental design

A field trial was conducted in a commercial winery vineyard in the region of Pydna, Greece (40° 30′ 0″ North, 22° 31′ 60″ East) planted with 15 years old *Vitis vinifera* L. cv. Syrah (clone 470 grafted on R110). The vineyard was established on an east-facing slope of 5%, and the vines were spaced 1.2 m within the row and 2.2 m between rows, while the rows were orientated W-E. The soil was characterized as sandy clay, while the regional climate has been classified as C*fa* according to Köppen’s classification (Kottek et al., 2006). Vine plants were trained according to a double cordon vertical shoot positioned (VSP) system with a trunk height of 1 m above ground and a canopy of 1.3–1.5 m over the fruiting wire. Since the establishment of the vineyard the Integrated Pest or Disease Management (IPM/IDM) principles were followed to combat pests and diseases and the use of conventional pesticides was limited.

The effects of six agronomic practices on *Aspergillus* Black rot disease incidence and grape carposphere microbiome were determined during two consecutive growing seasons (2019 and 2020). We followed a split-block experimental design with three (3) replicated blocks and nine (9) grapevines per treatment. Each block consisted of the following treatments: (a) defoliated (DF), (b) non-defoliated (NDF), (c) irrigated (IR), (d) non-irrigated (NIR), (e) high bunch density (HBD), and (f) low bunch density (LBD). Defoliation (DF) was performed prior to berry set as total removal of leaves of the first 6 nodes from the base of the shoot until bunches were completely exposed to sunlight, while non-defoliated (NDF) grapevines were used as a control. Irrigation treatments (IR) involved irrigation on 15 days intervals from berry set through harvest. Irrigation was applied by a drip irrigation system positioned on either side of the trunk, while entirely rainfed grapevines were used in the non-irrigated (NIR) treatment as a control and no irrigation was applied at any stage. In bunch density treatments, the high (HBD) and low bunch (LBD) density treatments were achieved with the variation of the number of buds retained at winter pruning (low/high respectively). More specifically, in HBD treatments, grapevines were spur-pruned to 12 buds per plant, while to achieve LBD, 24 buds were retained per plant. During the first growing season, all treatments were evaluated for their efficacy in reducing *Aspergillus* incidence on grape berries at harvest. In the second year, *Aspergillus* incidence was assessed at three phenological stages (according to the BBCH-scale for grapes): pea-sized berries (BBCH 75, ~2 months before harvest), veraison (BBCH 81, ~1 months before harvest) and harvest (BBCH 89); carposphere fungal and bacterial communities were determined at harvest via amplicon sequencing analysis.

### Culture-based determination of *Aspergillus* incidence on grape berries

A culture-based approach was followed to evaluate the impact of the applied agronomic practices on Black *Aspergilli* section *Nigri* incidence. In detail, asymptomatic grape bunches were collected at harvest (mid of August) in sterile sealed plastic bags and transferred in an ice-filled container to the laboratory, where they were processed. The isolation and counting of *Aspergillus* species was performed as previously described by Testempasis et al. (2022). Briefly, more than 300 berries (10 berries × 30 bunches) per treatment were randomly selected from each sample and surface disinfected (1 min in a 1% sodium hypochlorite solution). Afterward, berry samples were transversely cut and placed onto Petri dishes containing Dichloran Rose Bengal Chloramphenicol-Agar media (DRBC-agar based) and incubated at 27°C (dark) for seven (7) days. After the incubation period, Black *Aspergilli* section *Nigri* colonies were counted, and their occurrence frequency was calculated based on the number of obtained isolates per the total number of grapes. This analysis was performed in both years, while, in the second year, culture-based monitoring of *Aspergillus* occurrence was performed on each phenological stage of the grapevine.

### Berry quality parameters

Berry samples were used to analyze quality parameters, such as total soluble solids (TSS), pH, and titratable acidity (TA). More specifically, eight (8) grape bunches per treatment were sampled at the harvest stage, transported to the laboratory as mentioned previously, and immediately processed. Berry samples were manually pressed at room temperature, and the juice was used to determine the TSS using a manual refractometer (HI96841, Hanna instruments, United States); the pH with a pH-meter (HI2020-02, Hanna instruments, United States); and TA by titration with NaOH (0.1 N).

### Measurement of bunch density and water status

The bunch density was assessed at harvest. In total, 30 bunches were collected and evaluated per treatment (HBD and LBD), averaged across three replicates. For each bunch, measurements of: (a) cluster length (cm), (b) cluster mass (g), (c) rachis mass (g), (d) number of berries per cluster, (e) berry’s mass (g), and (f) cluster density index, were conducted. Berry mass and bunch density were calculated according to the following equations, as previously described by Tello and Ibanez (2014):


$$\text{Berr}y_{\text{mass}}=\frac{\text{Clustermass}-\text{Rachismass}}{\text{Numberofberries}}$$

$$\text{Berr}y_{\text{Density}}=\frac{\text{Clusterweight}}{\text{Clusterlengh}t^{2}}$$

Additionally, the water status of the irrigated and non-irrigated vines was estimated by measuring stem water potential (SWP) at harvest. Measurements were conducted at solar noon (12:00 to 13:30) using a pressure chamber, as previously described by Chone et al. (2001). Briefly, three (3) leaves from the inner part of the canopy of each vine were enclosed in plastic foil-covered bags for 90 min before the measurement to allow equilibration of water potential.

### Analysis of the grape carposphere microbiome

#### Samples processing and DNA extraction

Grapevine bunches were collected during the second experimental season (2020) at three phenological stages: pea-sized berry, veraison, and harvest. In detail, 30 intact and asymptomatic bunches were collected (10 bunches × three replicates/treatment/phenological stage) from both sides of each grapevine and carefully placed in sterile stomacher bags. Samples were transferred to the laboratory in a cooler with dry ice and immediately processed for extraction of its carposphere DNA as previously described by Vitulo et al. (2019), with slight modifications. In detail, 250 g of grapes were placed into a sterile 500 mL flask containing 250 mL of Phosphate Buffered Saline (PBS) isotonic solution. The microbial biomass on the surface of berries was detached via shaking for two (2) hours in a horizontal shaker (240 rpm) at room temperature. The suspension was then transferred to a sterile 250 mL centrifuge tube and centrifuged for 20 min at 4000 rpm. The supernatant was discarded and the pellet was subjected to DNA extraction using the DNeasy PowerSoil Pro Kit (Qiagen, United States) following the manufacturer’s protocol. DNA concentrations were determined using the Quan-iT kit with a Qubit Fluorometric device (Invitrogen, United States).

#### Amplicon sequencing analysis

The composition of the carposphere fungal and bacterial community was determined via multiplex amplicon sequencing of the ITS2 and the V4 region of the 16S rRNA gene, respectively. The amplification of the ITS2 region was performed using the primer set ITS7F/ITS4R (White et al., 1990; Ihrmark et al., 2012), while the bacteria 16 s rRNA gene was amplified with the primers 515F/806R (Parada et al., 2016) (Supplementary Table S1). Amplification, facilitating sample-wise multiplexing, and libraries preparation were performed according to our in-house protocol as described in detail by Vasileiadis et al. (2015). Briefly, a two-step amplification protocol was followed for sample indexing. The first amplification step included 28 cycles using the primer pairs mentioned previously, while the second amplification step consisted of 7 cycles using the same primer pairs with sample-specific 5′ overhangs (Supplementary Table S1). All PCR reactions were conducted with the Q5® HighFidelity DNA Polymerase (NEB, Massachusetts, United States), while amplicon libraries were cleaned using the NucleoMag NGS Clean up and Size Select kit (Macherey-Nagel, Duren, Germany). Sequencing was performed in a NovaSeq Illumina platform in Rapid Mode 2×250 paired-end in the Centre of Genomics, Biomedical Research Foundation Academy of Athens, Greece.

#### Bioinformatic analysis

Before the analysis, sequence de-multiplexing was performed using Flexbar 3.0.3 (Dodt et al., 2012). The dada2 package (v.1.18.0) (Callahan et al., 2016) of the R software version 4.0.5 (R Core Team, 2020) was used to quality-trim/filter the sequences, remove the chimerical sequences and generate the Amplicon Sequence Variant (ASVs) table. The reference databases UNITE ITS v.8.2 (Abarenkov, 2020) and Silva v.138 (McLaren, 2020) were used for the assembly and the taxonomic classification of the produced ASVs. The final analysis excluded ASVs that had not been classified at the Kingdom and Phylum levels. Additionally, the downstream analysis included only the ASVs that were taxonomically annotated with at least 80% bootstrap confidence in the indicated domain/kingdom taxa, as previously suggested by Kozich et al. (2013). The microbiome package (v.1.12.0) (Lahti and Shetty, 2012) was utilized to calculate the α-diversity indices of observed richness, Pielou’s evenness (Pielou, 1966), Shannon (Spellerberg and Fedor, 2003), and Inverse Simpson (Hill, 1973). Significant differences in ASVs differential abundance among treatments and plant phenological stages were assessed with the package Agricolae (v.1.3.3) (Mendiburu and Yaseen, 2020), using a parametric ANOVA or non-parametric Kruskal-Wallis analysis of variance followed by Tuckey’s or Fisher’s least-significant differences *post hoc* test, respectively. The β-diversity was addressed via canonical correspondence analysis (CCA) (ter Braak and Verdonschot, 1995) and redundancy analysis (RDA) (Israels, 1984) according to the first axis of detrended correspondence analysis (Leps and Šmilauer, 2003). Moreover, pairwise Adonis package v.0.0.1 (Arbizu, 2019) was used to calculate β-diversity’s permutation analysis of variance between the ASVs and the covariates of interest.

A network analysis was performed to identify the positive or negative co-occurrence networks of fungal and bacterial genera that participate in 10% of the samples with at least 1% relative abundance. In detail, Spearman Correlation tests were performed among microbial genera, and the data were used as an adjacency matrix for the weighted network analysis using the igraph v.1.2.6 package of R software (Csardi and Nepusz, 2006). On the resulted undirected networks, the adjacency matrix was filtered using the minimum spanning tree algorithm choosing the shortest possible combinations among tree nodes (Drago et al., 2017). Following that, the sub-community clusters were determined based on local densities, and the final network was plotted using the Fruchterman-Reingold layout (Fruchterman and Reingold, 1991). Network analysis was performed based on the analysis code as previously described by Bekris et al. (2022). The sequencing data were submitted to the Sequence Read Archive of NCBI with BioProject accession number (ID: PRJNA958223).

#### Statistical analysis

The occurrence frequency of *Aspergillus* isolates among treatments was compared with the *z*-test analysis. The significance level of all hypothesis testing procedures was predetermined at *p* = 0.05. Additionally, a *t*-test analysis was performed to determine the statistical significance of the examined quality parameters. Statistical analyses were conducted using SPSS v.20.0 software (Armonk, NY, United States). All the graphical presentations were created with GraphPad Prism (GraphPad Prism version 9.0.0 for Windows, GraphPad Software, San Diego, California, United States).

## Results

### Effect of agronomic practices on the *Aspergillus* incidence on grapevine berries

**Figure 1:**
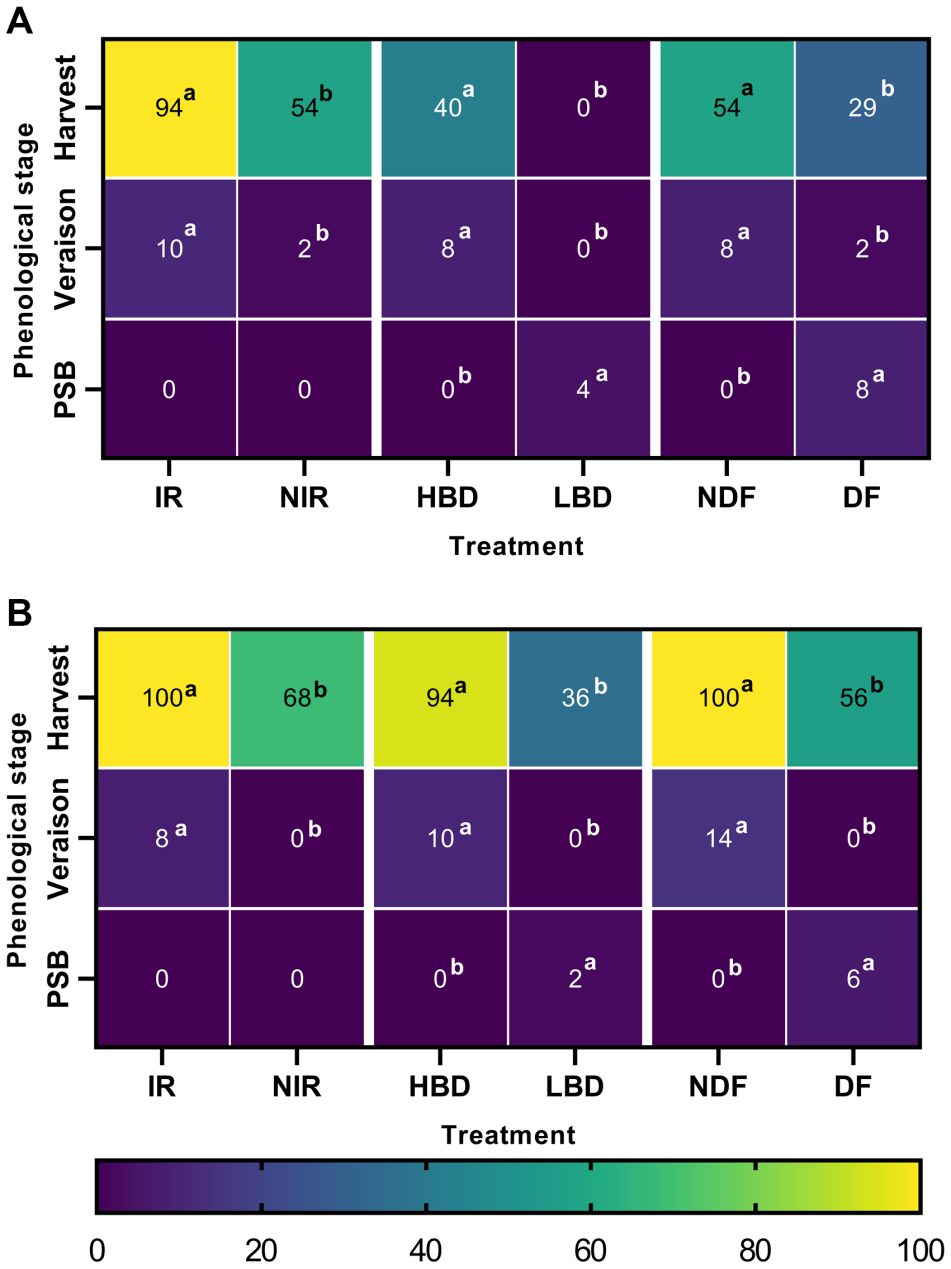
Heatmap graphical representation of *Aspergillus* spp. occurrence frequency (%) obtained from asymptomatic grape berries of all treatments (irrigated, IR; non-irrigated, NIR; high bunch density, HBD; low bunch density, LBD; defoliated, DF; non-defoliated, NDF), phenological stages (pea size berry, PSB; veraison and harvest), and year of sampling (**A**:2019; **B**:2020). Letters indicate the statistical significance between the treatments and their mock-pair on each phenological stage according to a series of *z*-test analyses (*p* = 0.05).

For both experimental years, we observed higher frequencies of *Aspergillus* occurrence, mainly at the harvest stage, followed by the veraison and pea-size berry stages (Figures 1A,B). In 2019, at the stages of pea size berry, veraison, and harvest, *Aspergillus* was detected at occurrence frequencies ranging from 0 to 8%, 0 to 10%, and 0 to 94%, respectively (Figure 1A). Treatment comparisons revealed significant differences (*p* < 0.05) in *Aspergillus* occurrence frequencies at harvest. In detail, *Aspergillus* was isolated at significantly higher occurrence frequencies (*p* < 0.05) in IR (94%) vs. NIR (54%), HBD (40%) vs. LBD (0%) and NDF (54%) vs. DF (29%) treatments (Figure 1A). Similar results were obtained during the second year of experimentation with *Aspergillus* being detected at the pea-size berry, veraison, and harvest stages at occurrence frequencies ranging from 0 to 6%, 0 to 14%, and 36 to 100%, respectively (Figure 1B). At harvest, *Aspergillus* showed significantly higher occurrence frequencies (*p* < 0.05) in the IR (100%) vs. NIR (68%), HBD (94%) vs. LBD (36%), and NDF (100%) vs. DF (56%) (Figure 1B).

### Effects of agronomic practices on grape berries quality and status

#### Effect of agronomic practices on berry quality parameters

The impact of agronomic practices on berry quality traits (TSS, TA, and pH) was evaluated at harvest (Table 1). In 2019, TSS significantly increased (*p* < 0.05) in the treatments of NIR (23.4 ^o^Brix) and DF (18.9 ^o^Brix) compared to IR (15.3 ^o^Brix) and NDF (16.1 ^o^Brix) respectively. However, no significant differences (*p* > 0.05) were evident in the TSS content between HBD and LBD (Table 1). TA and pH were only slightly affected by the tested agronomic practices (*p* > 0.05) with the sole exception of the significantly higher (*p* < 0.05) values in the LBD (7.3 g/L) vs. the HBD (6.2 g/L) treatment (Table 1). A similar pattern was observed in the second year: sugar content substantially increased (*p* < 0.05) in the NIR (22.1 ^o^Brix) and DF (18.4 ^o^Brix) treatments compared to IR (18.3 ^o^Brix) and NDF (16.3 ^o^Brix), respectively (Table 1) while TA significantly increased (*p* < 0.05) in the IR (8.5 g/L) and LBD (8 g/L) treatments compared to NIR (6 g/L) and HBD (7 g/L), respectively. No discernible pH variation among the tested treatments was found (Table 1).

**Table 1:** Average values of total soluble solids (^o^Brix) (TSS), titratable acidity (g/L of tartaric acid) (TA), and pH of grape berries at the harvest stage during the 2019 and 2020 growing seasons in response to the applied agronomic treatments.

Treatment	2019			2020		
	TSS	TA	PH	TSS	TA	PH
Irrigated	^*^15.3 ± 0.1^b^	7.6 ± 0.4^a^	3.2 ± 0.05^a^	18.3 ± 0.3^b^	8.5 ± 0.5^a^	3.3 ± 0.02^a^
Non-irrigated	23.4 ± 0.9^a^	6.9 ± 0.2^a^	3.4 ± 0.03^a^	22.1 ± 0.4^a^	6.0 ± 0.4^b^	3.6 ± 0.04^a^
Defoliated	16.1 ± 0.2^b^	8.8 ± 0.1^a^	3.1 ± 0.09^a^	16.3 ± 0.2^b^	7.9 ± 0.5^a^	3.3 ± 0.02^a^
Non-defoliated	18.9 ± 0.6^a^	8.1 ± 0.4^a^	3.1 ± 0.04^a^	18.8 ± 0.3^a^	8.6 ± 0.6^a^	3.4 ± 0.04^a^
High bunch density	18.5 ± 0.9^a^	6.2 ± 0.2^b^	3.3 ± 0.09^a^	19.5 ± 0.3^a^	7.0 ± 0.0^b^	3.4 ± 0.02^a^
Low bunch density	19.8 ± 0.8^a^	7.3 ± 0.2^a^	3.3 ± 0.09^a^	20.6 ± 0.5^a^	8.0 ± 0.6^a^	3.4 ± 0.02^a^

^*^ Mean value of TSS, TA, and pH measurements with respective standard deviations. Statistical analysis was performed between the treatment and its mock-pair using *t*-test analysis, while different letters indicate significant differences (*p* = 0.05).

#### Measurements of bunch density and water status

Significant differences between the HBD and LBD treatments were evident for the parameters studied (Supplementary Figures S1A,B). In both years, bunch density of the samples obtained from the HBD treatment (1.36 and 1.01 in 2019 and 2020 respectively) was significantly higher (*p* < 0.05) than the bunch density from LBD treatment (0.66 and 0.51), (Supplementary Figure S1). No noticeable differences were observed in berry mass measurements for both years with the values in HBD and LBD treatments ranging from 1.19 to 1.71 and 1.26 to 1.65 g, respectively (Supplementary Figure S1).

Measurements of SWP were performed on vines of IR and NIR treatments to verify the impact of the contrasting irrigation treatments on grapevine water status. Indeed, grapevine water status considerably differed between the IR and NIR treatments, with the SWP values for both ranging from −1.20 to −1.22 MPa and − 1.50 to −1.56 MPa, respectively (Supplementary Figures S2A,B), suggesting a higher water deficit for the NIR plants at harvest since SWP values at the range of −1.10 to −1.30 MPa are considered as a moderated water deficit, whereas values inferior to −1.50 MPa are representative for severe water deficit (Theocharis et al., 2021).

### Amplicon sequencing analysis

#### Microbial community composition and dynamics

Overall, 4,065,773 and 3,618,209 fungal and bacterial sequences were obtained. After quality control, an average of 37,998 ± 23,836 and 33,815 ± 16,156 of high-quality sequences per sample were attained for fungi and bacteria, respectively. Rarefaction curves reached a plateau in all samples, indicating that the sequencing provided adequate microbial diversity coverage. The sole exception was one sample from the NIR treatment at harvest (replicate C) which was excluded from the analysis due to poor sequencing quality (Supplementary Figure S3).

**Figure 2:**
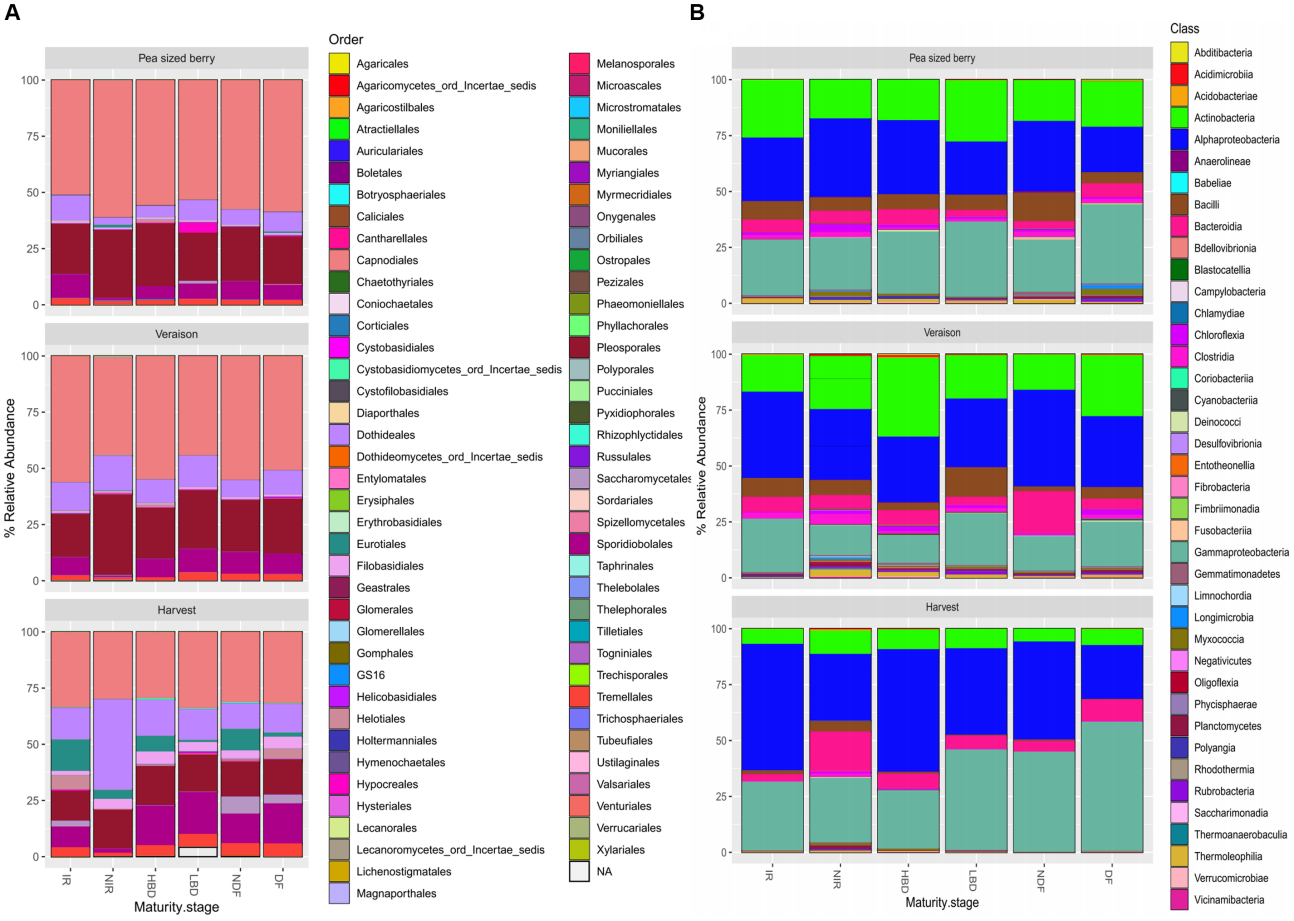
Stacked bar plots displaying the composition of the **(A)** fungal and **(B)** bacterial grape carposphere communities at the taxonomic level of Order and Class, respectively, across different phenological stages of grapevines (pea size berry, veraison, and harvest) and the agronomic treatments employed (irrigated, IR; non-irrigated, NIR; high bunch density, HBD; low bunch density, LBD; non-defoliated, NDF; defoliated, DF).

Regardless of the treatment, the fungal communities were dominated by fungi of the order *Capnodiales* (46.78% ± 11.24), followed by *Pleosporales* (22.00% ± 5.74), *Dothidiales* (11.90% ± 6.70), *Sporidiobolales* (9.28% ± 5.34), *Eurotiales* (3.94% ± 2.22) and *Tremellales* (3.04% ± 1.65) (Figure 2A). Interestingly, at the harvest stage we observed a considerable increase in the relative abundance of *Eurotiales*, where *Aspergillus* species belongs, in the treatments NDF (9.62% ± 1.12) vs. DF (1.90% ± 0.31), HBD (7.05% ± 0.16) vs. LBD (0.96% ± 0.07), and IR (14.08% ± 0.92) vs. NIR (4.04% ± 1.38) (Figure 2A).

The carposphere bacterial microbiome was dominated by Gamma-proteobacteria, Alpha-proteobacteria, Actinobacteria, and Bacilli, with a mean relative abundance of 37.89% ± 12.7, 36.89% ±12.9, 16.99% ± 11.35 and 4.05% ± 5.22, respectively (Figure 2B).

### The effect of agronomic practices and phenological stage on the α-diversity of carposphere microbial communities

**Figure 3:**
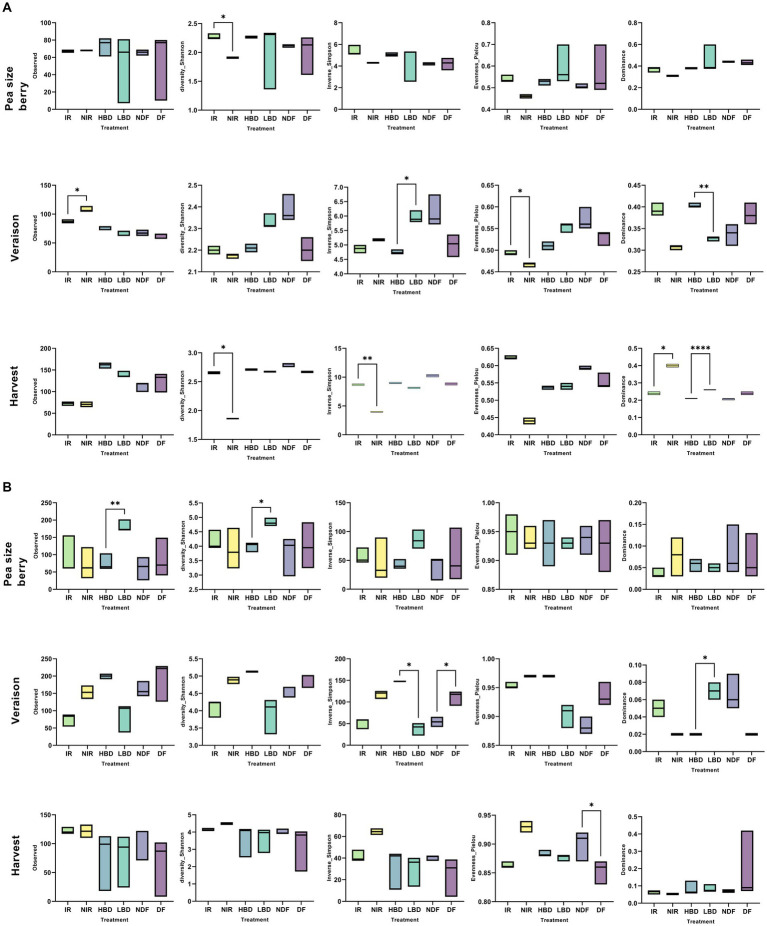
The α-diversity indices of the fungal **(A)** and bacterial **(B)** community in grape berries collected from the different treatments (irrigated, IR; non-irrigated, NIR; high bunch density, HBD; low bunch density, LBD; defoliated, DF; non-defoliated, NDF), at the different phenological stages (Pea size Berry, PSB; Veraison and Harvest). Statistical analysis was performed using Sidak’s multiple comparisons test, while asterisks (*p* < 0.05*, *p* < 0.01**, *p* < 0.001****) indicate significant differences.

The analysis of variance (two-way ANOVA) of the two main factors (phenological stage and treatments) was addressed to determine if they significantly affect the α-diversity indices of the fungal and bacterial communities (Supplementary Table S2). Analysis revealed a significant interaction of treatment and phenological stage (*p* < 0.05) regarding the α-diversity indices (Supplementary Table S2). We first looked at the effects of different agronomic practices on the fungal α-diversity at each growth stage. At the pea size berry stage, agronomic practices did not induce any significant effect (*p* > 0.05) on any of the α-diversity indices measured, except of Shannon diversity which was significantly higher (*p < 0.05*) at the IR vs. NIR (Figure 3A). At veraison, we noted (i) a significantly higher richness but significantly lower Pielou’s evenness (*p < 0.05*) in the NIR vs. IR treatment and (ii) a significantly lower Inverse Simpson (*p < 0.05*) but a significantly higher dominance (*p < 0.01*) in the HBD vs. LBD treatment (Figure 3A). At harvest, we observed (i) a significantly reduced Shannon and Inverse Simpson indices (*p < 0.05*) in the NIR vs. IR treatment and (ii) a significant lower dominance of LBD (*p < 0.001*) and NIR (*p < 0.05*) compared to the HBD and IR treatments (Figure 3A). Regarding the α-diversity of bacteria, at the pea size berry stage, we noted significantly higher richness (*p* < 0.01) and Shannon diversity (*p* < 0.05) values in the LBD vs. HBD (Figure 3B). A reduction (*p* < 0.05) in Shannon diversity values in NDF and LBD treatments was noticed at veraison compared to their respective mock pairs (Figure 3B). Furthermore, a significantly higher (*p* < 0.05) dominance was observed in LBD vs. HBD. At harvest, agronomic practices did not induce significant effects (*p* > 0.05) on the α-diversity of bacteria except of the Pielou’s evenness, which was significantly reduced (*p* < 0.05) in DF vs. NDF (Figure 3B).

When looking across phenological stages we noted a significant increase (*p < 0.05*) in richness, Shannon, and inverse Simpson diversity indices at harvest, while the exact reverse pattern was evident for Dominance where significantly lower values were noted at harvest (Supplementary Figure S4A). Regarding bacteria, we observed a significantly higher (*p < 0.05*) richness, Shannon and inverse Simpson values at veraison, unlike Evenness and Dominance which showed significantly higher and lower values (*p < 0.05*) respectively at harvest (Supplementary Figure S4B).

### The effect of agronomic practices and phenological stage on the β-diversity of the fungal and bacterial communities on grape carposphere

The β-diversity of the fungal and bacterial community was significantly affected by the phenological stage of the grapevine (*p* = 0.001) with this factor contributing 36.2 and 4.7% of the total variance, respectively, (Supplementary Figures S5A,B). Pairwise comparison of the fungal and bacterial communities RDA fitted values between the different phenological stages revealed significant differences (*p* < 0.05) (Supplementary Figures S5A,B).

**Figure 4:**
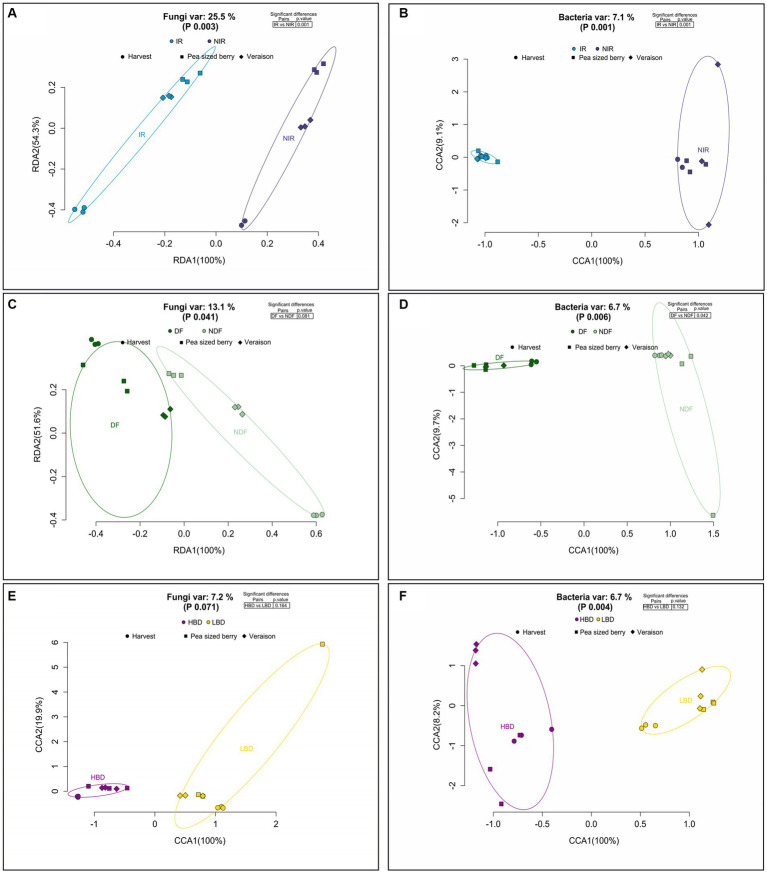
Redundancy analysis (RDA) and Canonical Correspondence Analysis (CCA) of the fungal **(A)** and bacterial **(B)** communities of grapes carposphere. Samples were ordinated based on the different treatment comparisons (IR vs. NIR; NDF vs. DF; HBD vs. LBD) **(A–F)**, regardless of the grapevine phenological stage. Inserted tables present the comparisons of the microbial communities among the different treatments **(A–F)**.

We further looked at the effect of the applied agronomic practices on the β-diversity of the fungal and bacterial carposphere communities. The performed treatments considerably affected the β-diversity of the fungal community (Figures 4A,C) with the exception of bunch density treatments (*p* = 0.071) which did not induce any significant effect on fungal communities (Figure 4E). In particular, the agronomic practices of irrigation (IR and NIR) (*p* = 0.001) and defoliation (DF and NDF) (*p* = 0.041), considerably affected the b-diversity of the fungal community as the RDA revealed that irrigation and defoliation explained the 25.5 and 13.1% of the total variance, respectively (Figures 4A,C). In addition, a pairwise comparison of the fungal community RDA fitted values between the IR vs. NIR and DF vs. NDF treatments revealed significant differences (*p* < 0.05). Further, the performed treatments significantly affected the bacterial community, as CCA performed for the treatments of irrigation (*p* = 0.001) (Figure 4B), defoliation (*p* = 0.006) (Figure 4D), and bunch density (*p* = 0.004) (Figure 4F) at all phenological stages explained the 7.1, 6.7, and 6.7% of the total variance, respectively (Figures 4B,D,H). Pairwise comparisons of the bacteria community CCA fitted values between the IR vs. NIR (Figure 4B) and DF vs. NDF (Figure 4D) treatments showed significant differences (*p* < 0.05), while no significant differences (*p* > 0.05) were observed in the comparison of HBD vs. LBD treatments (Figure 4F).

### The effect of agronomic practices on specific fungal genera at harvest

**Figure 5:**
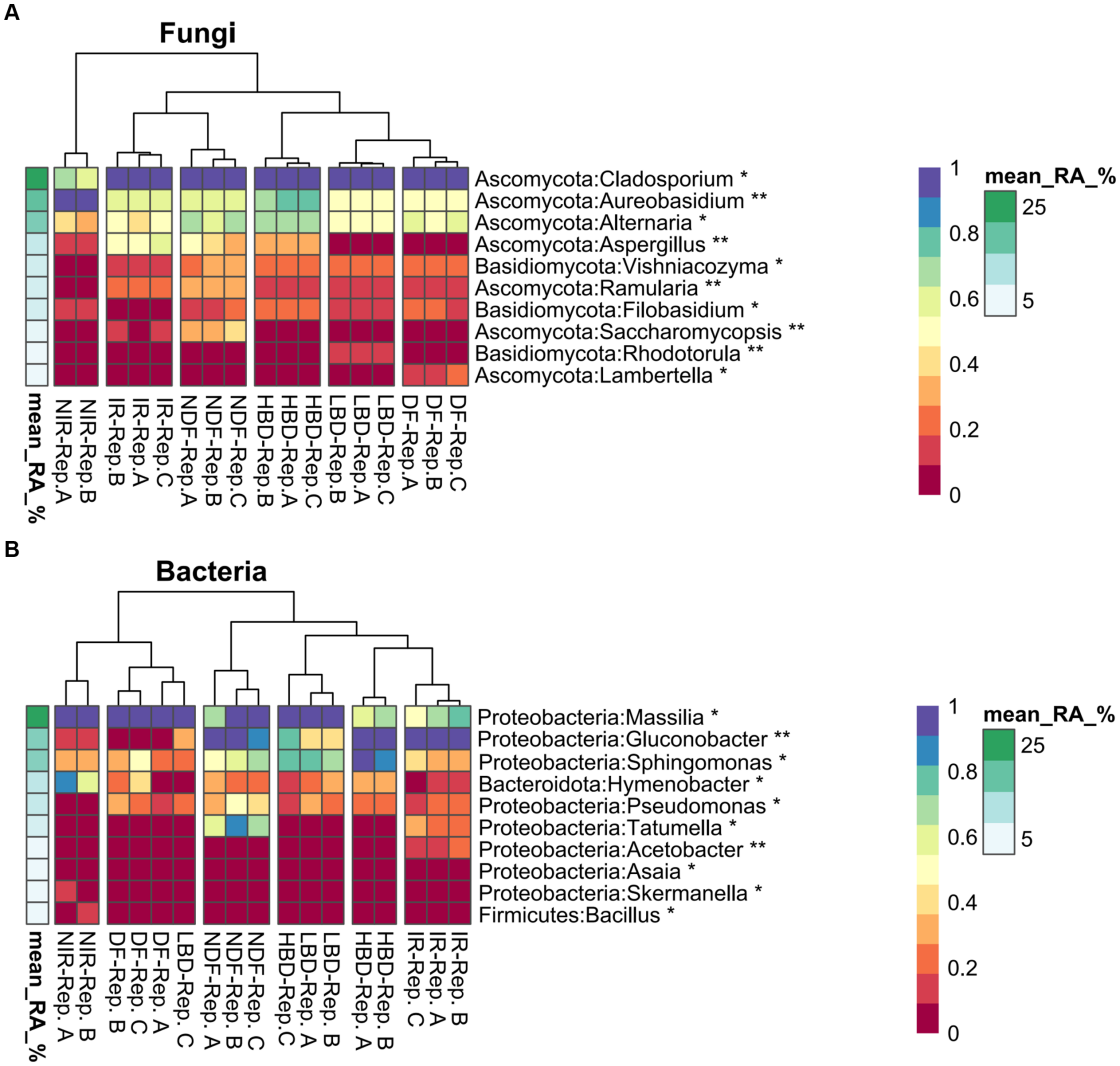
Differential abundance (DA) heatmaps showing fungal **(A)** and bacterial **(B)** agglomerated ASVs at the genus level. Asterisks indicate significant differences (*p* < 0.05*. *p* < 0.01**) in the relative abundance among the treatments at the harvest stage. Presented DA values were standardized between 0 and 1 for ease of visualization. Experimental treatments with individual replicates are noted below each heatmap, while the differential abundance and the mean relative abundance color scale bar were presented on the side of each heatmap.

Based on the high occurrence of *Aspergillus* spp., mostly at the harvest stage, bacterial and fungal communities ASVs were agglomerated at genus level and screened for significant effects stemming by the different agronomic treatments (Figure 5). Regarding fungi, ASVs belonging to *Cladosporium* were dominated in all treatments, except NIR which was dominated by ASVs of the genus *Aureobasidium* (Figure 5A). ASVs of the genus *Alternaria* were associated with the treatments HBD and NDF, while ASVs of the genus *Aspergillus* were strongly associated with treatments HBD, NDF, and IR (Figure 5A). We further verified the effect of the performed agronomic practices on the frequency of occurrence of *Aspergillus* spp., by using data from the amplicon sequencing analysis. The occurrence frequency of *Aspergillus* ASVs in the IR, HBD and NDF treatments was significantly higher (*p* < 0.05) compared to the NIR, LBD and DF treatments, respectively, (Supplementary Figures S6A–C).

Regarding bacteria, ASVs belonging to *Massilia* were favored in the treatments IR and HBD compared to NIR and LBD (Figure 5B). Likewise, ASVs belonging to *Gluconobacter* were significantly associated with treatments IR, HBD, and NDF. Finally, ASVs that belong to *Pseudomonas* was disfavored in the NIR treatments, whereas ASVs belonging to *Tatumella* were favored in IR and NDF treatments (Figure 5B).

### Network co-occurrence analysis of epiphytic grapes microbial communities

**Figure 6:**
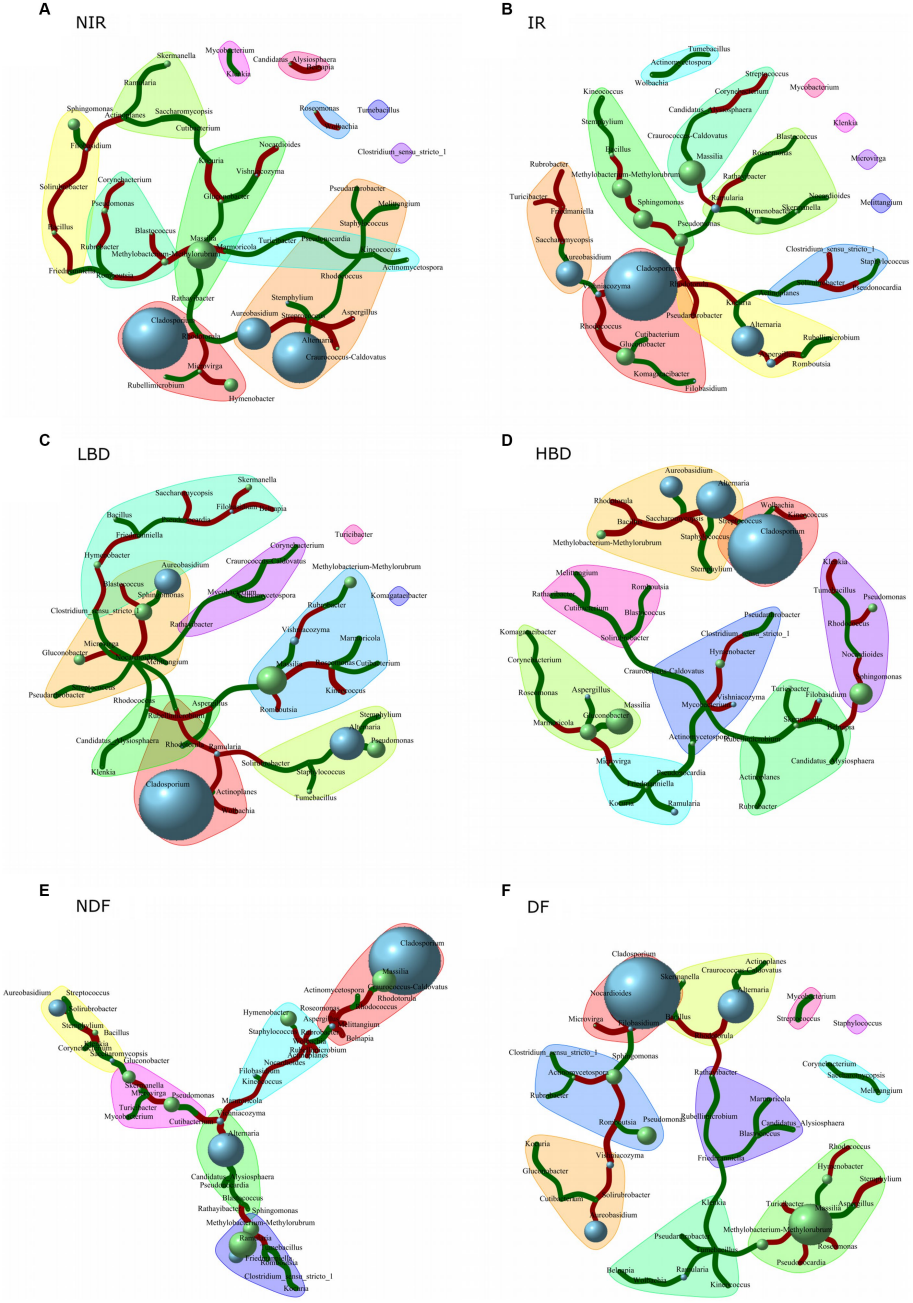
Network co-occurrence analysis of the fungal and the bacterial grape caropshere community in the different agronomic treatments: **(A)** NIR, non irrigated; **(B)** IR, irrigated; **(C)** LBD, low bunch density; **(D)** HBD, high bunch density; **(E)** NDF, non defoliated; and **(F)** DF, defoliated treatment. Network analysis was performed with the fungal and bacterial genera whose relative abundance was >1% in 10% of the samples. Blue and green bubbles indicate the relative abundance of each microbial genera, (the higher the size of the bubble the higher the microbial relative abundance) respectively, while green and red links demonstrate significant positive and negative co-occurrence patterns, respectively, between the linked microorganisms. The width of the line is a measure of the level of the co-occurrence correlation between the linked microorganisms, as the greater width of the line implies a higher correlation between the co-occurrence of the microorganisms.

Network co-occurrence analysis was employed to identify significant positive or negative co-occurrence patterns between *Aspergillus* and members of the epiphytic fungal and bacterial communities that are either systematic among all treatments or associated with specific treatments (Figure 6). We observed a negative co-occurrence pattern of *Aspergillus* with *Alternaria* and *Romboutsia* in the IR treatment and with *Streptococcus* in the NIR treatment (Figures 6A,B). A significant positive co-occurrence pattern was observed between *Aspergillus* and *Gluconobacter* in the HBD treatment, while *Aspergillus* was positively correlated with *Rhodotorula* and *Massilia* and negatively correlated with *Rhodococcus* in the LBD treatment (Figures 6C,D). Finally, a significant positive co-occurrence pattern was noted between *Aspergillus* – *Wolbachia* in the DF and between *Aspergillus* – *Massilia* in the NDF treatment. In contrast, a significant negative co-occurrence pattern was observed between *Aspergillus* and several bacterial genera like *Melitangium, Rhodococcus, Belnapia, Roseomonas* in DF, and between *Aspergillus* and *Stemphylium* in the NDF treatment (Figures 6E,F).

## Discussion

It is now well documented that agricultural practices manipulating the canopy of grapevines or the compactness of grape bunches may influence the microclimate within grapevines by changing the aeration, illumination, temperature, and air humidity and establishing less favorable conditions (reduced light, low ventilation, and high air humidity) for fungal infections (De Bem et al., 2015). In our study we report for the first time on the impact of such management practices (defoliation, irrigation, and bunch density) on the establishment of *Aspergillus*, as causal agents of bunch rot, and widely on the carposphere fungal and bacterial communities at three different phenological stages (pea size berry, veraison, and harvest).

By following a culture-based approach, we observed a reduced incidence of *Aspergillus* spp. in DF compared to NDF grapevines. In line with this observation, Duncan et al. (1995) reported that leaf removal reduced the population of many fungi on berries, such as *Aspergillus* spp., *Penicillium* spp., and *Cladosporium* spp. They suggested that leaf removal decreased the vigor of fungal conidia due to their higher exposure to ultraviolet radiation, aeration, and reduced humidity.

The effects of irrigation on numerous physiology and quality traits of vines and grapes have been investigated extensively over the past years (Garganese et al., 2016; Thomidis et al., 2016; Coniberti et al., 2018; Alatzas et al., 2021; Bassoi et al., 2021). Most of these studies have focused on plant physiology, while only a few have assessed the impact of irrigation on disease development (Thomidis et al., 2016; Coniberti et al., 2018). According to our findings, water deficit (NIR) decreased the occurrence frequency of *Aspergillus* compared to irrigation treatments (IR). Likewise, researchers found a lower incidence of bunch rot disease in a recent study testing the under-trellis cover crops to minimize water availability and vegetative growth (Coniberti et al., 2018). Climate change has affected viticulture due to rising temperatures, changes in rainfall patterns, and extreme events like droughts and heatwaves, leading to reduced water availability (Santos et al., 2020), and requiring more efficient use of water applications (Chaves et al., 2010). In regions with seasonal droughts, strategic vineyard irrigation may contribute to the increase of the yield, the vegetative growth and maintain the quality of wine production. However, irrigation may promote excessive vegetative growth, negatively impacting berries quality and susceptibility to fungal diseases (Bravdo et al., 1985; Dry and Loveys, 1998). On the other hand, deficit irrigation may increase the yield, improve the quality, and increase the tolerance of grapevine to bunch rot pathogens by triggering strong immune responses (Santos et al., 2005; Chaves et al., 2010). More specifically, drought stress tolerance in grapevines was involved in the activation of polyamine oxidation and phenylpropanoid pathway and consequently reduced their susceptibility to bunch rot pathogens (Hatmi et al., 2013; Alatzas et al., 2021).

Another significant factor investigated in our study was the impact of bunch compactness on Black Aspergilli frequency. We showed that their abundance was considerably higher in the grape bunches with higher density than loose bunches. Comparable results have been mentioned in the past by several researchers for other pathogens causing bunch rots like *B. cinerea* (Vail and Marois, 1991; Vail et al., 1998; Valdes-Gomez et al., 2008; Hed et al., 2009). Low bunch compactness improves the fungicide coverage and reduces the relative humidity levels inside the bunch due to enhanced inner aeration (Tello et al., 2015). Moreover, loose bunches show less physical damage caused by berry-to-berry contact, which may result in the appearance of microscopic cracks in berries cuticles (Hed et al., 2011; Becker and Knoche, 2012; Molitor et al., 2012).

We subsequently explored the composition of the grapevine carposphere microbiome and the effects of the different agronomic practices on its composition. The grape carposphere fungal communities was dominated by Ascomycetes (*Capnodiales, Pleosporales, Dothidiales*, and *Eurotiales*) and Basidiomycetes (*Sporidiobolales, Tremellales*), in line with previous reports (Gao et al., 2019; Wei et al., 2022). Similarly, the grape carposphere bacterial communities were dominated by Gamma-proteobacteria, Alpha-proteobacteria, Actinobacteria, and Bacilli, in accord with previous studies (Portillo et al., 2016; Wei et al., 2022).

Phenological stage influenced the α-diversity of bacteria and fungi colonizing the grapevine carposphere. Fungal species richness and diversity increased along the grape growing stages, which has been attributed to the accumulation of sugars on grapes that favor fungal colonization (Renouf et al., 2005; Martins et al., 2014; Liu et al., 2020). In contrast, the α-diversity of bacteria showed an increasing richness and diversity at veraison. The variable temporal patterns of α-diversity could be attributed to the differences in the content of growing grapes that favor different microbial groups, high bacterial diversity at veraison in accordance with the higher acid content and lower sugar content of berries at this stage, and higher fungal diversity at harvest where the sugar content facilitate the establishment of a diverse and rich fungal community (Setati et al., 2012; Liu et al., 2020; Wei et al., 2022).

The β-diversity of the grapevine’s associated microbiota was considerably affected by the applied agronomic practices and plant development stage. No Irrigation (NIR) vs. irrigation (IR) induced significant changes in the composition of the carposhpere fungal and bacterial communities. In support of our findings, Wei et al. (2022) suggested that the water status of vineyards could strongly influence the composition of the grapevine microbiome. Similarly, defoliation (DF) significantly altered the composition of the carposphere microbiome compared to NDF. This is in accordance with previous studies by Liu et al. (2020) who suggested that such changes on the grapevine microbiota by defoliation may be associated with the greater exposure of grape berries to a range of abiotic stress conditions (UV radiation, temperature, and humidity).

Beyond treatments, the carposphere microbiota was strongly affected by the phenological stage studied. In line with our findings, Ding et al. (2021) showed that the carposphere fungal and bacterial communities on Ecolly grapes considerably differed at each phenological stage. This temporal change in the composition of the carposphere microbiota along the growing season is a common feature of several different plant hosts (Rastogi et al., 2012; Grady et al., 2019). It has been suggested that the ripening process induces strong changes in the nutrient composition of grapes, reciprocating changes in the community of bacteria and fungi colonizing the surfaces of growing grapes (Liu et al., 2020; Ranade et al., 2021).

We further investigated the effect of agronomic practices on the abundance of the dominant members of the fungal and bacterial community on grapevine carposphere to identify members whose relative abundance showed treatment–specific patterns. We noted that *Aspergillus* and *Alternaria* were both favored in the IR, HBD and NDF treatments. Regarding *Aspergillus*, their higher relative abundance on the carposphere of grapes in those treatments is in accordance with their higher occurrence in the culture-dependent measurements on grape berries. Members of *Aspergillus* and *Alternaria* have been identified as common pathogens of grapevines favored by conditions like humidity and damaged berries which prevail in those treatments (Belli et al., 2007). *Cladosporium* was the dominant member of the carposphere in most treatments, but it seemed to be outcompeted by *Aureobasidium* when no irrigation was applied. Members of this genus are known biocontrol agents against several fungal pathogens like *Aspergillus* and their occurrence in grapevine carposphere has been commonly reported (Dimakopoulou et al., 2008; Zhang et al., 2019; Ding et al., 2021). Regarding bacteria, members of the genus *Massilia* and *Gluconobacter* were associated with IR and HBD treatments. Previous studies by Bokulich et al. (2013), showed that the relative abundance of *Gluconobacter* is negatively correlated with the TSS content of Chardonnay musts. This is in line with our study, where *Gluconobacter* species showed higher relative abundance in the HBD and IR treatments which were characterized by significantly lower TSS content of grapes.

We further examined the presence of significant co-occurrence patterns of *Aspergillus* with other fungal and bacteria genera and how these are affected by the different treatments employed. Interestingly, in the treatments of NIR, LBD, and DF, we observed negative co-occurrence patterns between *Aspergillus* and several bacterial genera like *Streptococcus* (NIR), *Rhodococcus* (LBD and DF) and *Melitangium* (DF), that carry antifungal properties. *Streptococcus* species could degrade *Aspergillus* spp. mycotoxins (Wiseman and Marth, 1981; Coallier-Ascah and Idziak, 1985), while in the human pathosystem, *Streptococcus pneumoniae* was found to disrupt *Aspergillus fumigatus* biofilm through hydrogen peroxide secretion (Iwahashi et al., 2020). Likewise, bacteria of the genus *Rhodococcus* are able to inhibit the growth and mycotoxin production of several *Aspergillus* species (Barbey et al., 2013; Pylak et al., 2020; de la Huerta-Bengoechea et al., 2022). Species belonging to the genus *Melitangium* are well-known producers of the antifungal compound melithiazole A (Hyun et al., 2016). Modifications in the carposphere microbiome’s composition carry significant implications for both disease management and the overall quality of grapes. Specific microbial species contribute to the sensory characteristics of grapes, such as flavor and aroma (e.g., *Saccharomyces cerevisiae*) (Swiegers et al., 2005), while some microbial groups play crucial roles in suppressing pathogenic organisms (e.g., *Bacillus subtilis, B. amyloliquefaciens, Ampelomyces quisqualis*, and *Aureobasidium pullulans*) (Pertot et al., 2017a). Even though the presence of microbial communities inside and outside plant tissues is a significant factor in disease development, there is limited understanding of how microbial consortia could effectively be used to prevent diseases (Lamichhane and Venturi, 2015). We suggest that these consistent negative co-occurrence patterns between the above epiphytic bacteria and *Aspergillus* might be part of a concerted natural attenuation mechanism of the grape microbial community to combat infestations by members of the grapevine pathobiome like *Aspergillus*. Still dedicated follow-up studies, using a combination of culture-dependent approaches (*in vitro* and *in planta*) along with metatranscriptomic analysis of the grapevine-associated microbiota, are needed to verify these interactions.

## Conclusion

Our study constitutes the first attempt, using a combination of culture-based and omic approaches, to unravel the impact of phenological stages and three commonly used agronomic practices (leaf removal, bunch compactness, and irrigation) on *Aspergillus* occurrence and on the overall grapevine carposphere microbiome. Our two-year field study showed that defoliation in the fruiting zone, grape bunch loosening caused by an increased retained number of buds at pruning, and absence of irrigation reduced the occurrence frequency and relative abundance of *Aspergillus* on grape berries and strongly affected the composition of the carposphere fungal and bacterial communities. Further studies using shotgun meta-omic approaches will explore the pathogenic and general functional potential of the grapevine carposphere as affected by the different agronomic practices tested.

## Data availability statement

The raw amplicon sequences and accompanying metadata have been deposited in the National Center for Biotechnology Information Sequence Read Archive under the BioProject accession number PRJNA958223.

## Author contributions

SIT: Conceptualization, Data curation, Formal analysis, Investigation, Visualization, Writing – original draft, Writing – review & editing, Methodology. CP: Data curation, Investigation, Methodology, Visualization, Writing – review & editing. ST: Investigation, Writing – review & editing. PK: Investigation, Methodology, Writing – review & editing. SK: Conceptualization, Methodology, Supervision, Writing – review & editing. DK: Conceptualization, Supervision, Writing – review & editing. GK: Conceptualization, Methodology, Supervision, Writing – review & editing.
