# Supplementary material for: Vineyard practices reduce the incidence of Aspergillus spp. and alter the composition of carposphere microbiome in grapes (Vitis vinifera L.)
Source: Front Microbiol. 2023 Nov 24;14:1257644. doi: 10.3389/fmicb.2023.1257644 (PMC10704512; doi:10.3389/fmicb.2023.1257644)
Supplement: Supplementary file 1 [file Data_Sheet_1.docx]

**Table S1**. Primers, sequences, and thermocycling conditions used for amplicon sequencing analysis of the bacterial and fungal communities

**^1^** The sample index (sequential Ns) and linker (bold and underlined letters) prior to the extension bases in the forward or reverse primer are indicated.

**^2^** Indicates the number of cycles performed in the second step of the two-step amplification process where the indexed primers were used.


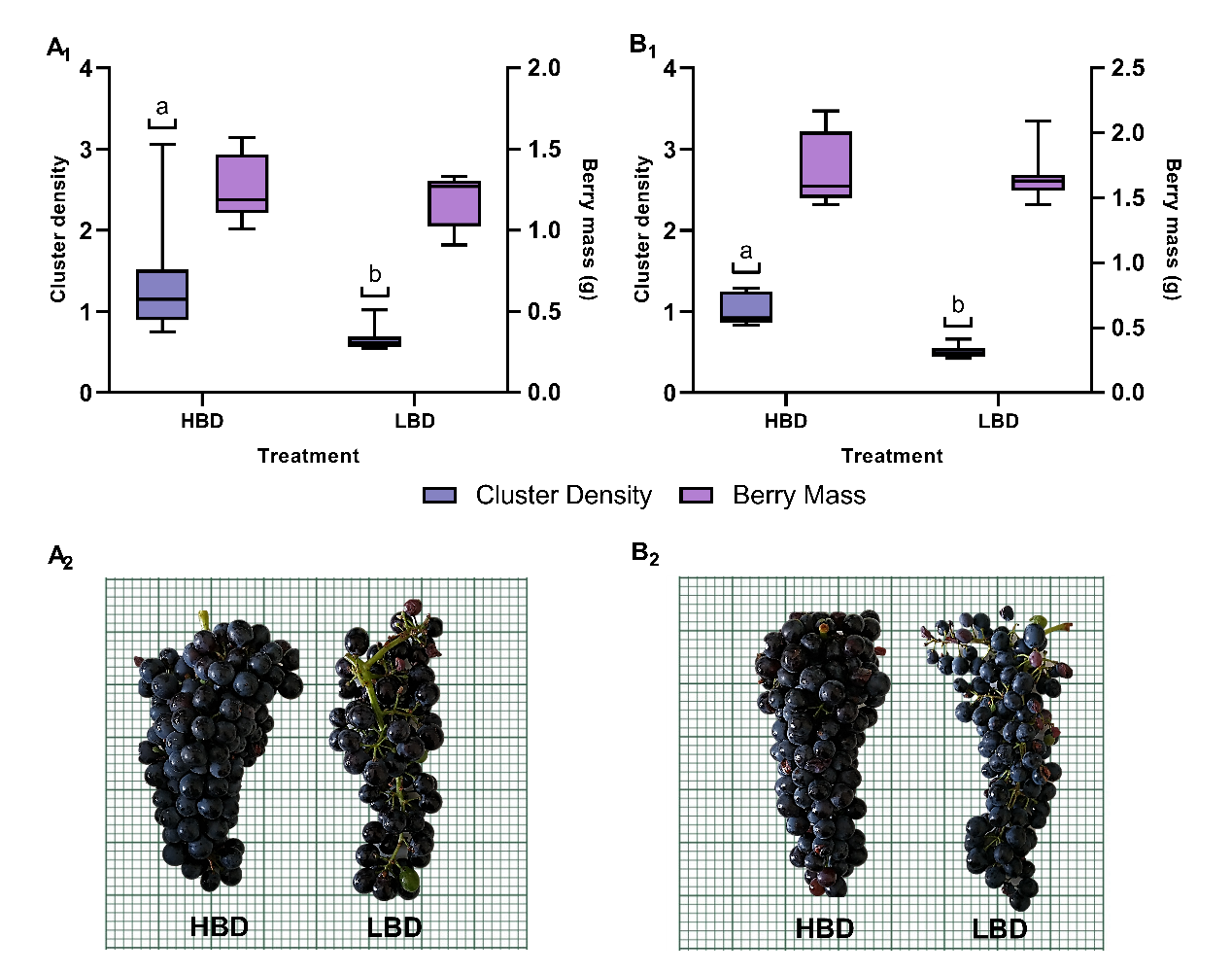


**Figure S1**. Measurements of Cluster density and Berry mass (g) of samples collected at the harvest stage from the HBD and LBD treatments for two consecutive years (2019 and 2020). **A_1_ & B_1_****:** box and whiskers graphs present the minimum, average, and maximum values of cluster density and berry mass for 2019 and 2020, respectively. **A_2_ & B_2_:** demonstrate the effect of HBD and LBD on produced grape clusters for 2019 and 2020. Statistical analysis was performed between the treatment and mock-pair using *t*-test analysis, while different letters indicate significant differences (*P*=0.05).


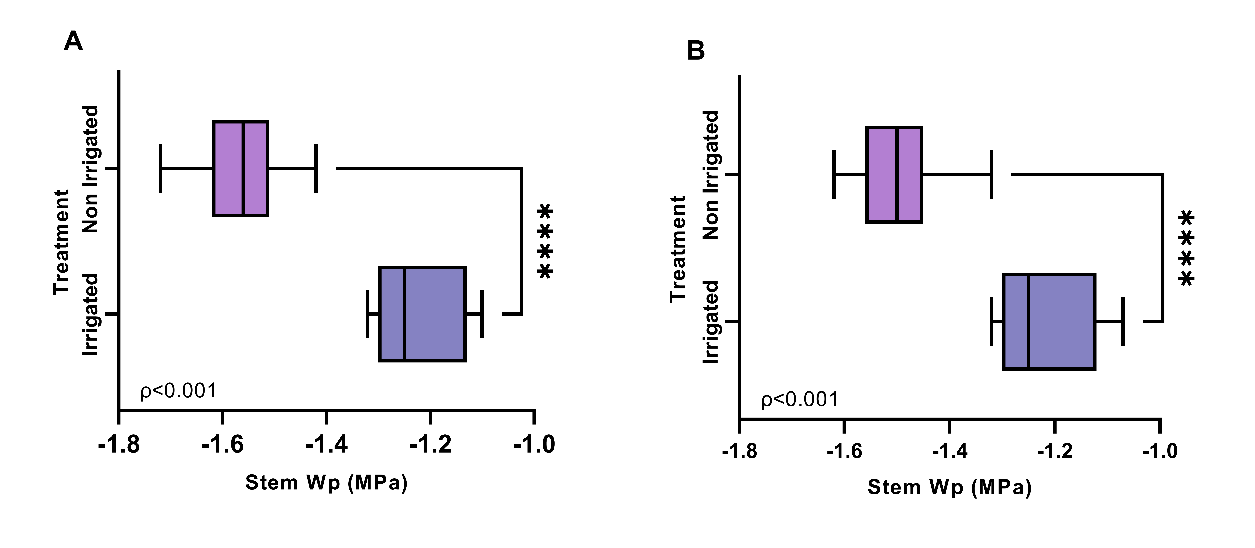


**Figure S2**. Measurements of Stem water potential (SWP) at the harvest stage of irrigated and non-irrigated vines. A & B: box and whiskers graphs present the minimum, average and maximum values of stem water potential (MPa) for 2019 and 2020, respectively. Statistical analysis was performed between the treatment and mock-pair using *t*-test analysis, while asterisks indicate significant differences (P=0.05).


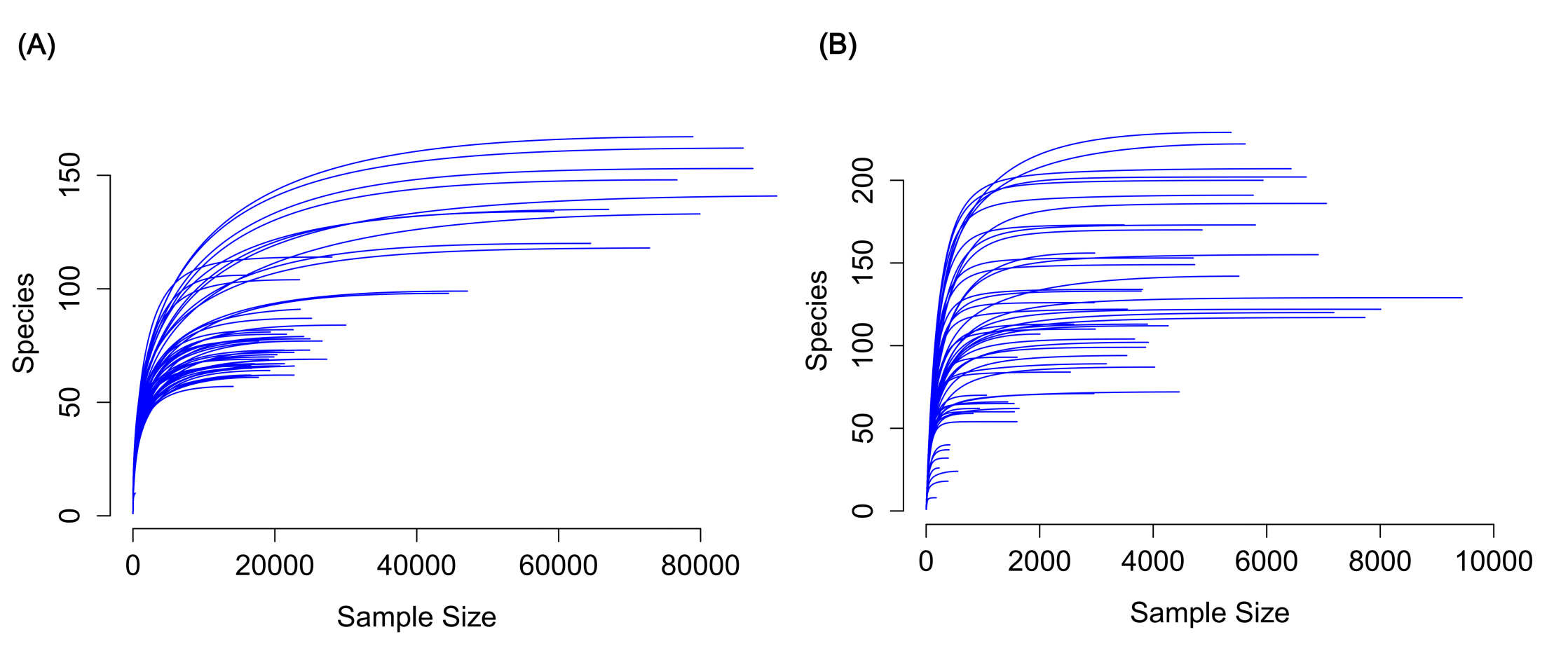


**Figure S3**. Rarefaction curves of samples obtained of grape berries carposphere and analyzed for their (A) fungal and (b) bacterial microbiome.

**Table S2**. Analysis of variance (two-way ANOVA) of different parameters (treatments and phenological stages) on fungal (A) and bacterial (B) α-diversity indices (Observed, Diversity Shannon, Inverse Simpson, Pielou’s eveness, and Dominance).

| 1. **Fungi** | | | | | | |  | 1. **Bacteria** | | | | | | |
| --- | --- | --- | --- | --- | --- | --- | --- | --- | --- | --- | --- | --- | --- | --- |
| **Observed** | | | | | | |  | **Observed** | | | | | | |
| **Comparisons** | **DF^1^** | **Sum Sq^2^** | **Mean Sq^3^** | **F value^4^** | **Pr (>F)^5^** | **Sign.^6^** |  | **Comparisons** | **DF** | **Sum Sq** | **Mean Sq** | **F value** | **Pr (>F)** | **Sign.** |
| Treatment | 5 | 4118 | 824 | 3.40 | 0.01 | * |  | Treatment | 5 | 3006 | 601 | 0.42 | 0.83 | ns |
| Phenological stage | 2 | 24743 | 12371 | 51.10 | 0.00 | *** |  | Phenological stage | 2 | 32129 | 16065 | 11.20 | 0.00 | *** |
| Treatment * Phenological stage | 10 | 18967 | 1897 | 7.84 | 0.00 | *** |  | Treatment * Phenological stage | 10 | 75354 | 7535 | 5.25 | 0.00 | *** |
| **Diversity Shannon** | | | | | | |  | **Diversity Shannon** | | | | | | |
| **Comparisons** | **DF** | **Sum Sq** | **Mean Sq** | **F value** | **Pr (>F)** | **Sign.** |  | **Comparisons** | **DF** | **Sum Sq** | **Mean Sq** | **F value** | **Pr (>F)** | **Sign.** |
| Treatment | 5 | 0.91 | 0.18 | 6.86 | 0.00 | *** |  | Treatment | 5 | 0.76 | 0.15 | 0.51 | 0.77 | ns |
| Phenological stage | 2 | 2.10 | 1.05 | 39.69 | 0.00 | *** |  | Phenological stage | 2 | 5.03 | 2.52 | 8.33 | 0.00 | ** |
| Treatment * Phenological stage | 10 | 0.90 | 0.09 | 3.40 | 0.00 | ** |  | Treatment * Phenological stage | 10 | 7.83 | 0.78 | 2.59 | 0.02 | * |
| **Inverse Simpson** | | | | | | |  | **Inverse Simpson** | | | | | | |
| **Comparisons** | **DF** | **Sum Sq** | **Mean Sq** | **F value** | **Pr (>F)** | **Sign.** |  | **Comparisons** | **DF** | **Sum Sq** | **Mean Sq** | **F value** | **Pr (>F)** | **Sign.** |
| Treatment | 5 | 23.23 | 4.65 | 20.13 | 0.00 | *** |  | Treatment | 5 | 8243.00 | 1649.00 | 4.51 | 0.00 | ** |
| Phenological stage | 2 | 134.64 | 67.32 | 291.61 | 0.00 | *** |  | Phenological stage | 2 | 21407.00 | 10703.00 | 29.31 | 0.00 | *** |
| Treatment * Phenological stage | 10 | 42.60 | 4.26 | 18.45 | 0.00 | *** |  | Treatment * Phenological stage | 10 | 28679.00 | 2868.00 | 7.85 | 0.00 | *** |
| **Pielou's eveness** | | | | | | |  | **Pielou's eveness** | | | | | | |
| **Comparisons** | **DF** | **Sum Sq** | **Mean Sq** | **F value** | **Pr (>F)** | **Sign.** |  | **Comparisons** | **DF** | **Sum Sq** | **Mean Sq** | **F value** | **Pr (>F)** | **Sign.** |
| Treatment | 5 | 0.07 | 0.01 | 9.52 | 0.00 | *** |  | Treatment | 5 | 0.01 | 0.00 | 5.24 | 0.00 | ** |
| Phenological stage | 2 | 0.01 | 0.04 | 2.60 | 0.09 | ns |  | Phenological stage | 2 | 0.03 | 0.01 | 29.50 | 0.00 | *** |
| Treatment * Phenological stage | 10 | 0.04 | 0.04 | 2.82 | 0.01 | * |  | Treatment * Phenological stage | 10 | 0.02 | 0.00 | 3.79 | 0.00 | ** |
| **Dominance** | | | | | | |  | **Dominance** | | | | | | |
| **Comparisons** | **DF** | **Sum Sq** | **Mean Sq** | **F value** | **Pr (>F)** | **Sign.** |  | **Comparisons** | **DF** | **Sum Sq** | **Mean Sq** | **F value** | **Pr (>F)** | **Sign.** |
| Treatment | 5 | 0.00 | 0.00 | 0.88 | 0.51 | ns |  | Treatment | 5 | 0.01 | 0.00 | 0.99 | 0.44 | ns |
| Phenological stage | 2 | 0.01 | 0.10 | 90.44 | 0.00 | *** |  | Phenological stage | 2 | 0.02 | 0.01 | 3.77 | 0.03 | * |
| Treatment * Phenological stage | 10 | 0.12 | 0.01 | 11.12 | 0.00 | *** |  | Treatment * Phenological stage | 10 | 0.04 | 0.00 | 1.29 | 0.27 | ns |

**^1^** DF: degrees of freedom for each variable

**^2^** Sum Sq: Sum of squares

**^3^** Mean Sq: Mean sum of squares

**^4^** F value: test statistic from F test

**^5^** Pr(>F) is the p-value of the F statistic

**^6^** Sign: Significance code indicates significant differences (P<0***, p<0.001**, p<0.05*, p>0.05^ns^)


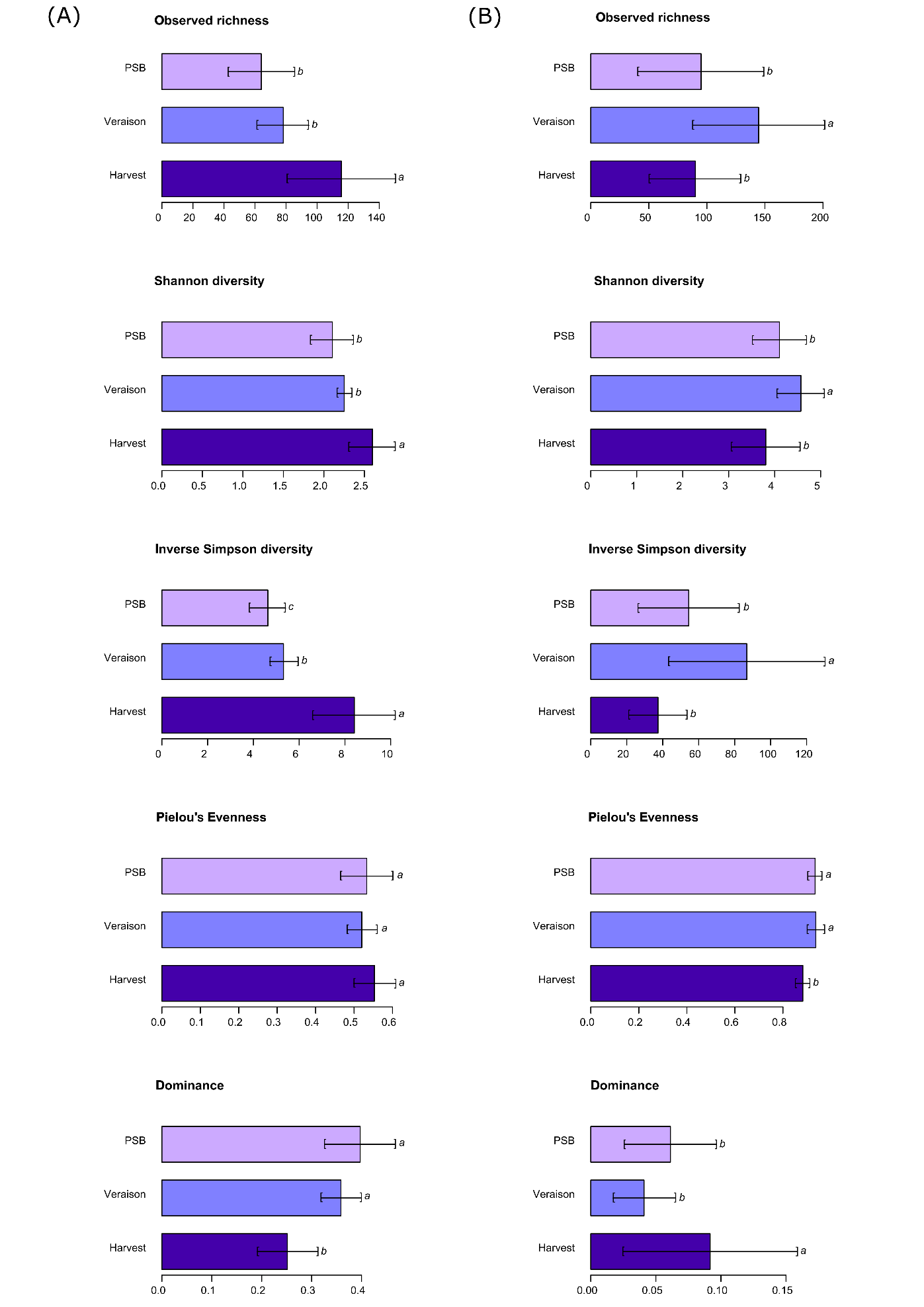


**Figure S4**. The α-diversity indices of the fungal (A) and bacterial (B) carposphere community of grapes at the different phenological stages (Pea size berry; PSB, Veraison, and Harvest). Statistical analysis was performed using Kruskal-Wallis test, while different letters indicate significant differences (p=0.05).


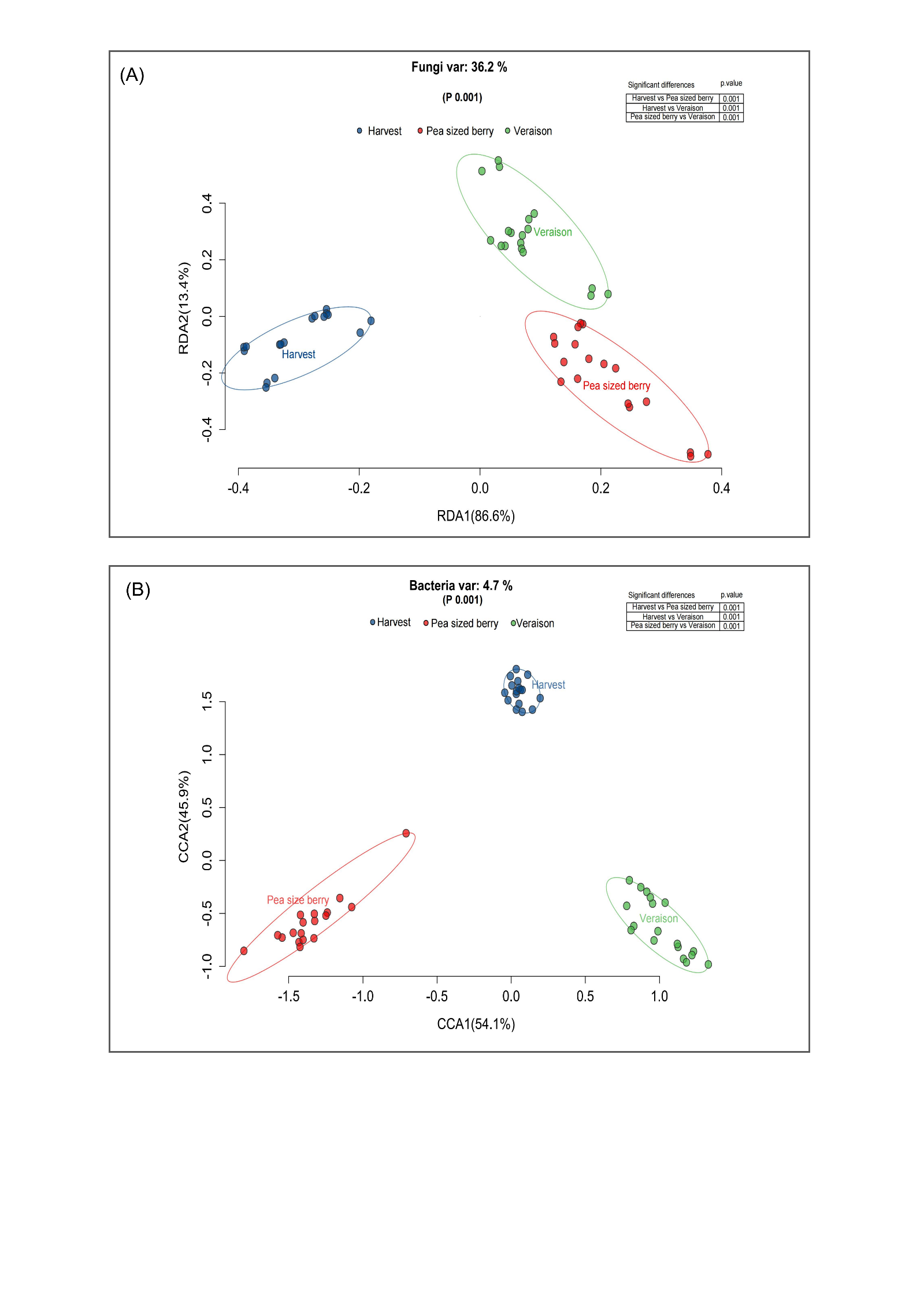


**Figure S5**. Redundancy analysis (RDA) and Canonical Correspondence Analysis (CCA) of the fungal (A) and bacterial (B) communities of grapes carposphere. Samples were ordinated based on grapevine phenological stage (Pea size berry, Veraison, and Harvest) (A-B), regardless of the treatments. Inserted tables present the comparisons of the microbial communities among the different phenological stages (A-B).


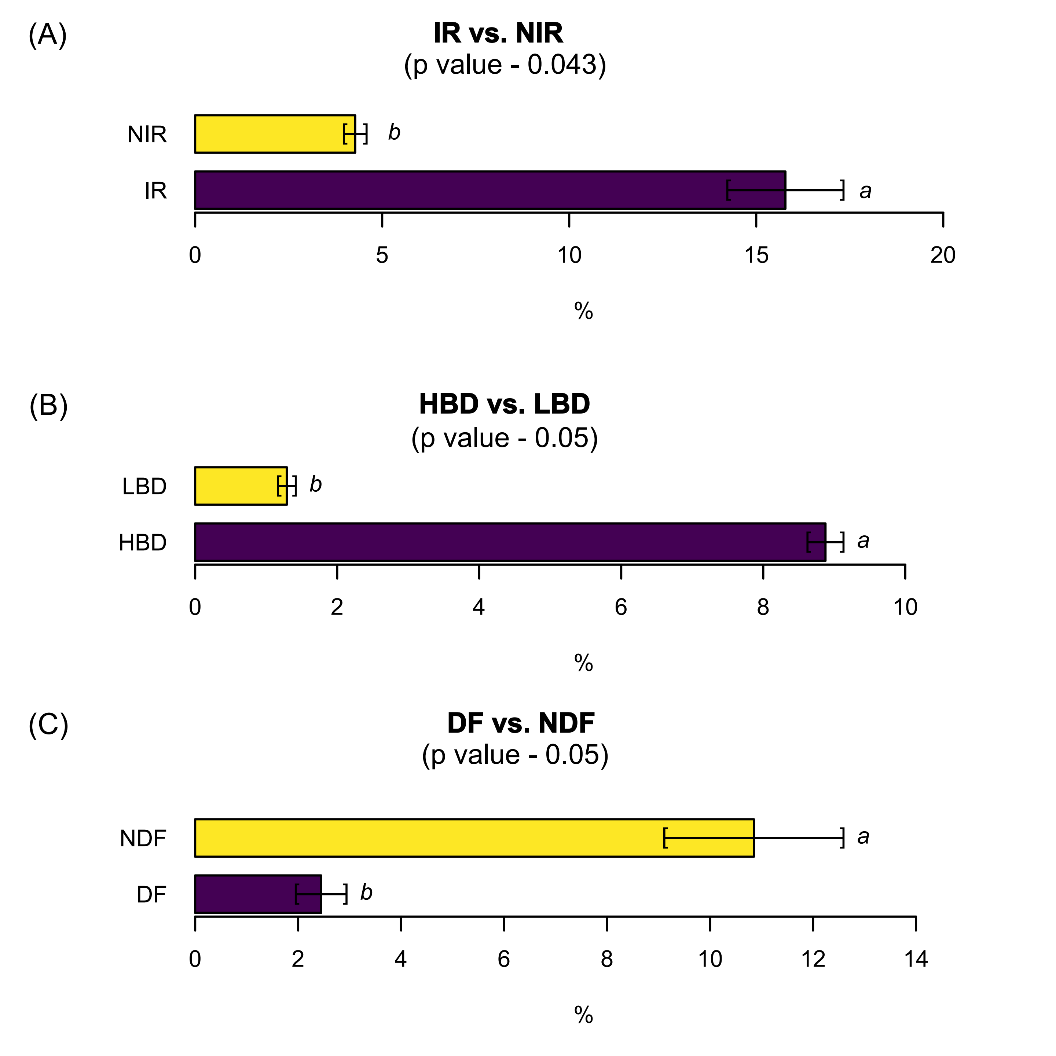


**Figure S6**. Relative abundance bar plots showing the relative abundance of *Aspergillus* spp. ASVs in the different treatment comparisons (A) IR vs. NIR, (B) HBD vs. LBD, and (C) NDF vs. DF at harvest. Bars designated by different letters are significantly different (*P*<0.05).
